# Protein kinase GCN2 mediates responses to glyphosate in *Arabidopsis*

**DOI:** 10.1186/s12870-014-0378-0

**Published:** 2015-01-21

**Authors:** Isabel Faus, Ana Zabalza, Julia Santiago, Sergio G Nebauer, Mercedes Royuela, Ramon Serrano, Jose Gadea

**Affiliations:** Instituto de Biología Molecular y Celular de Plantas (IBMCP), Universitat Politécnica de València (UPV)-Consejo Superior de Investigaciones Científicas (CSIC). Ciudad Politécnica de la Innovación (CPI), Ed. 8E. C/ Ingeniero Fausto Elio s/n, 46022 Valencia, Spain; Departamento de Producción Vegetal, Universitat Politécnica de València (UPV), Camino de Vera s/n, 46022 Valencia, Spain; Departamento de Ciencias del Medio Natural, Universidad Pública de Navarra, Campus Arrosadía, 31006 Pamplona, Spain

**Keywords:** Glyphosate, Gcn2, Transcriptomic, Shikimate, Translation, Herbicide

## Abstract

**Background:**

The increased selection pressure of the herbicide glyphosate has played a role in the evolution of glyphosate-resistance in weedy species, an issue that is becoming a threat to global agriculture. The molecular components involved in the cellular toxicity response to this herbicide at the expression level are still unidentified.

**Results:**

In this study, we identify the protein kinase GCN2 as a cellular component that fosters the action of glyphosate in the model plant *Arabidopsis thaliana*. Comparative studies using wild-type and *gcn2* knock-out mutant seedlings show that the molecular programme that the plant deploys after the treatment with the herbicide, is compromised in *gcn2*. Moreover, *gcn2* adult plants show a lower inhibition of photosynthesis, and both seedlings and adult *gcn2* plants accumulate less shikimic acid than wild-type after treatment with glyphosate.

**Conclusions:**

These results points to an unknown GCN2-dependent factor involved in the cascade of events triggered by glyphosate in plants. Data suggest either that the herbicide does not equally reach the target-enzyme in a *gcn2* background, or that a decreased flux in the shikimate pathway in a *gcn2* plants minimize the impact of enzyme inhibition.

**Electronic supplementary material:**

The online version of this article (doi:10.1186/s12870-014-0378-0) contains supplementary material, which is available to authorized users.

## Background

Since its introduction in 1974, glyphosate has become the world most widely used herbicide, especially after the emergence of transgenic resistant crops in 1996. In 2007, more than 80% of the transgenic crops worldwide were engineered to be glyphosate-resistant [1]. An increase in the application frequency of the herbicide has, however, played a role in the evolution of glyphosate-resistance in weedy species, an issue that is becoming a threat to global agriculture. At least three of the ten most conspicuous weeds have evolved resistant to glyphosate after one decade of transgenic crops [2]. In this scenario, understanding the molecular responses to glyphosate can be useful for future biotechnological approaches.

Resistance to herbicides can be achieved by alterations in the gene encoding the target protein, causing a reduction in the efficacy of the herbicide (target-site resistance) or by any other mechanism independent of the target enzyme (non-target-site resistance, NTSR) [3]. NTRS, that includes decreased herbicide penetration into the plant, decreased rate of herbicide translocation and increased rate of herbicide sequestration/metabolism, has been reported to be the most widespread type of resistance to glyphosate [4]. Although several of these mechanisms have been proposed [5,6], the molecular components involved are still unidentified.

Glyphosate affects plants systemically after application to the leaf surface. The phytotoxic symptoms develop slowly, plant death requiring days or weeks depending on the dose applied [7]. Inhibition of its target enzyme 5-enolpyruvylshikimate-3-phosphate-synthase (EPSPs; EC 2.5.1.19), inhibits the shikimate pathway, leading to a shortage in aromatic amino acids, quinones and cofactor biosynthesis. This is considered by some authors as the main cause of glyphosate toxicity, consistent with the slow development of symptoms [7]. In contrast, others consider that shikimate accumulation, due to a decrease in feedback inhibition through the pathway, leads to an energy drain imposed by a utilization of one phosphoenolpyruvate (PEP) molecule and one ATP molecule for every molecule of shikimate-3-phosphate accumulated and 3 ATP’s for every NADPH [8,9]. Herbicides inhibiting amino acid biosynthesis also induce non-target indirect effects, as proteolysis and an increase in free amino acids [10]. The last omics studies [11,12] reveal that the full picture of molecular disturbances after EPSP inhibition is complex and far from being totally understood. One of the biochemically best characterized cellular events after glyphosate treatment is the rapid shutdown of photosynthesis [13]. Cessation of carbon fixation, decrease in chlorophyll content and electron transport has been reported to occur soon after herbicide application. This impairment is not only a metabolic perturbation but involves gene expression and protein contents. Turfgrass and soybean plants exposed to glyphosate repressed most genes related to photosynthesis [14,15] and repression of photosynthetic proteins was observed in rice after glyphosate treatment [11]. Besides photosynthesis, other cellular processes such as cell cycle [16] cell motility (in bacteria) [12], cell death and redox homeostasis [11] are directly or indirectly affected by glyphosate.

In yeast, amino acid starvation is followed by activation of the protein kinase GCN2 by uncharged tRNAs. This enzyme phosphorylates the α subunit of eukaryotic translation initiation factor 2 (eIF2α), inhibiting the conversion of eIF2γ-GDP to eIF2γ -GTP, preventing further cycles of translation initiation and suppressing protein synthesis [17]. Phosphorylation of eIF2α not only causes a general reduction of protein synthesis, but initiates the selective translation of GCN4, an mRNA containing short open reading frames (ORF) upstream of the long, protein-coding ORF. Its translation upon amino acid starvation produces a transcription factor that activates amino acid biosynthesis genes, helping the cell to recover from the stress. This regulatory response is known as general amino acids control (GAAC) [18].

Plants contain a GCN2 homologue kinase that complements the *gcn2* yeast mutant strain, indicating that the GAAC response could be also operating in plants [19]. In *Arabidopsis* plants with the aromatic amino acids biosynthesis impaired as imposed by glyphosate treatment, eIF2α is phosphorylated, and this phosphorylation is GCN2-dependent, as it was abolished in an insertion line in the *GCN2* gene [20]. Phosphorylation of eIF2α by GCN2 has been observed under several abiotic stresses [20,21], and a GCN2-dependent translational arrest has been observed after treatment with chlorsulfuron, an inhibitor of branched amino acid biosynthesis [21]. Despite these data, the importance of GCN2 pathway as a regulatory mechanism in plants is still under debate [22-24]. The potential homologous gene of the yeast GCN4 has not been found in *Arabidopsis*, and little evidence was found for the involvement of AtGCN2 in the regulation of expression of amino acid biosynthesis genes, suggesting that this kinase could be playing another role than just regulating amino acid biosynthesis [25]. The role of GCN2 in the plant response to glyphosate remains elusive.

The transcriptional response to glyphosate has been determined in the bacteria *Escherichia coli* [12] in the grass *Festuca* [14] and in soybean [15] and in all cases a large number of genes change in expression in response to the herbicide. In the model plant *A. thaliana*, however, only a few genes (sixteen) were detected as modulated by glyphosate in leaves [26]. This discrepancy, and the unexpected behavior of aAtGCN2 in the regulation of amino acid biosynthetic genes, prompted us to reinvestigate the transcriptional response to glyphosate in *Arabidopsis* and to give some insights into the role of GCN2 in the triggering of this response.

In this study we show that glyphosate treatment triggers a complex transcriptional response in *Arabidopsis* plants. Surprisingly, many of these responses are not triggered or are altered in a *gcn2* mutant line. We also show that shikimate accumulation in *gcn2* plants after herbicide treatment is lower than in wild-type plants. All these results indicate that GCN2 is an important factor in the response of plants to glyphosate and that this protein kinase fosters the action of the herbicide by some unknown mechanism.

## Results

### Glyphosate treatment causes a dramatic shift of the *Arabidopsis* transcriptome

To get a better understanding of the transcriptional changes that glyphosate treatment provoke in *Arabidopsis* plants, 16-day-old plantlets were submerged in 200 μM glyphosate for 1 min, and gene expression was analysed 6 h after treatment. None of the glyphosate-derived phenotypic effects in the plant were visible at this time. Compared to mock-treated plants of the same age, more than 200 gene ontology (GO) biological processes were altered with a threshold applied of 0.05 (adjusted p-value, Methods). As expected, GO categories such as response to drug (adj. p-value 0.004) or multidrug transport (adj. p-value 0.003) are enriched in the glyphosate treated sample. In fact, 10 out the 30 most induced genes are ABC transporters, glutathione-transferases or glycosyltransferases [see Additional file 1], enzymes known to be involved in the detoxification of herbicides [27]. ABC transporter expression has been recently correlated with glyphosate resistance in horseweed [28]. Also expected, *Arabidopsis* plants respond to glyphosate activating the metabolism of aromatic amino acids (AAA) (0.1×10^−5^), including some genes in the biosynthetic pathways, but also genes of the secondary metabolism pathways that have AAAs as precursors (such as lignin, auxins, phenylpropanoid and others). For instance, genes coding for arogenate dehydratase, which catalyze the last step of phenylalanine (Phe) biosynthesis, and anthranilate synthase, phosphoribosylanthranilate transferase, indole-3-glycerol-phosphate synthase and trypthophan synthase, involved in trypthophan (Trp) biosynthesis, are all induced by glyphosate. Moreover, genes coding for phenylalanine-ammonia-lyase, (involved in phenylpropanoids biosynthesis, using Phe as a precursor), CYP79B3, or CYP71B15, (involved in IAA and camalexin biosynthesis, respectively, both using Trp as a precursor) are also induced. In addition, a plethora of genes belonging to many defense-related categories, including wounding, heat, oxidative, osmotic, cold, and biotic stresses are also induced, suggesting that not only specific, but also general responses, are triggered by a particular stress. Finally, categories such as aging or programmed cell death are enriched among the induced genes, revealing that, as early as 6 h after herbicide treatment, the plant could be already committed to die (see Table 1 and Additional file 2).

**Table 1:** **Selected categories enriched in Arabidopsis seedlings after glyphosate treatment (for a complete list, see Additional file** **2** **)**

**Selected GO categories enriched in glyphosate-induced genes**		**Selected GO categories enriched in glyphosate-repressed genes**	
**GO biological process**	**Adj. P-value**	**GO biological process**	**Adj. P-value**
Proteolysis	6.74×10^−24^	Photosynthesis	7.06×10^−29^
Defense response	5.60×10^−12^	Microtubule-based movement	3.71×10^−9^
Response to wounding	8.68×10^−11^	Photosynthetic electron transport chain	1.26×10^−8^
Response to bacterium	7.88×10^−10^	Porphyrin biosynthetic process	3.89×10^−7^
Aromatic amino acid metabolic process	0.000001169	Cell division	0.0000295
Response to osmotic stress	0.0000400	Electron transport chain	0.000260
Cell death	0.001031	Translation	0.00127
Response to oxidative stress	0.00318	Fixation of carbon dioxide	0.00468
Multidrug transport	0.00373	Regulation of cell size	0.00614
Response to drug	0.00483	Cell growth	0.011

Selected categories of a Gene Set Enrichment Analysis using Fatiscan (Medina et al. [51]) on the expression values of 16-days-old Arabidopsis wild-type Landsberg erecta seedlings treated with glyphosate, as described in Methods. For a complete list of GO categories (biological process) with an adjusted p-value lower than 0.05, see Additional file 2.

Most remarkable, the herbicide-treated plants show a dramatic down-regulation of the photosynthesis (7.06×10^−29^ adj. p-value), including chlorophyll biosynthesis (3.89×10^−07^), electron transport chain (1.26×10^−08^), and, to a lesser extent, CO_2_ fixation (4.68×10^−03^) (Table 1 and Additional files 3 and 4), a fact that was already observed at a later time-point (5 days after treatment) by Cebeci and Budak [14], in turtgrass. As examples, protochlorophyllide oxidoreductase (AT1G03630), involved in chlorophyll biosynthesis, is repressed more than 9 times; and PsbQ (AT1G14150) and PSB29 (AT2G20890), part of the PSII, are repressed more than 4 times. Interestingly, categories related to translation (including genes for the translation elongation factor EF1B (AT1G64510) and some ribosomal proteins (AT2G24090, AT1G07320 or AT1G48350, among others, repressed more than 4 times), growth and cell division (including genes encoding cyclin A1;1 (AT1G44110) or cyclin D1;1(AT1G70210), repressed more than 4 times) are also down-regulated [see Additional file 4], probably indicating that plant metabolism is being reprogrammed to cope with the stress situation [29].

### The presence of the protein kinase Gcn2 is necessary for the deployment of early cellular responses after glyphosate treatment

GCN2 is a protein kinase that phosphorylates the α subunit of the eIF2 translation initiation factor, a key regulatory mechanism that arrest general protein synthesis and allows the re-stablishment of homeostasis in eukaryotes after several stress conditions [17]. In *Saccharomyces cerevisiae*, GCN2 is activated under amino acid starvation and triggers a general translational arrest but also promotes the selective translation of the transcription factor GCN4. This factor activates transcription of several hundred genes, including those involved in amino acid biosynthesis [17]. As glyphosate is known to block AAA biosynthesis, we wanted to know whether a similar mechanism was operating in plants, and also to identify other cellular responses to glyphosate eventually regulated by GCN2. We compared the transcriptome of 16-day-old *Arabidopsis* wild-type seedlings with that of *gcn2* mutant seedlings of the same age [20], both at 6 h after glyphosate treatment. As described in Zhang et al. [20] and shown in Additional file 5: Figure S2, *gcn2* plants are phenotypically indistinguishable from wild-type plans at this and all stages of growth. Previously, we had compared the transcriptome of the same plants under normal conditions and determined that only 24 genes were changing their expression using the selected criteria. [see Additional files 1 and 3]. As shown in Additional file 6: Figure S1, and as reported by Zhang et al. [20], eIF2α phosphorylation indicates that, after 6 h treatment, GCN2 kinase is activated in wild-type plants.

**Figure 1:**
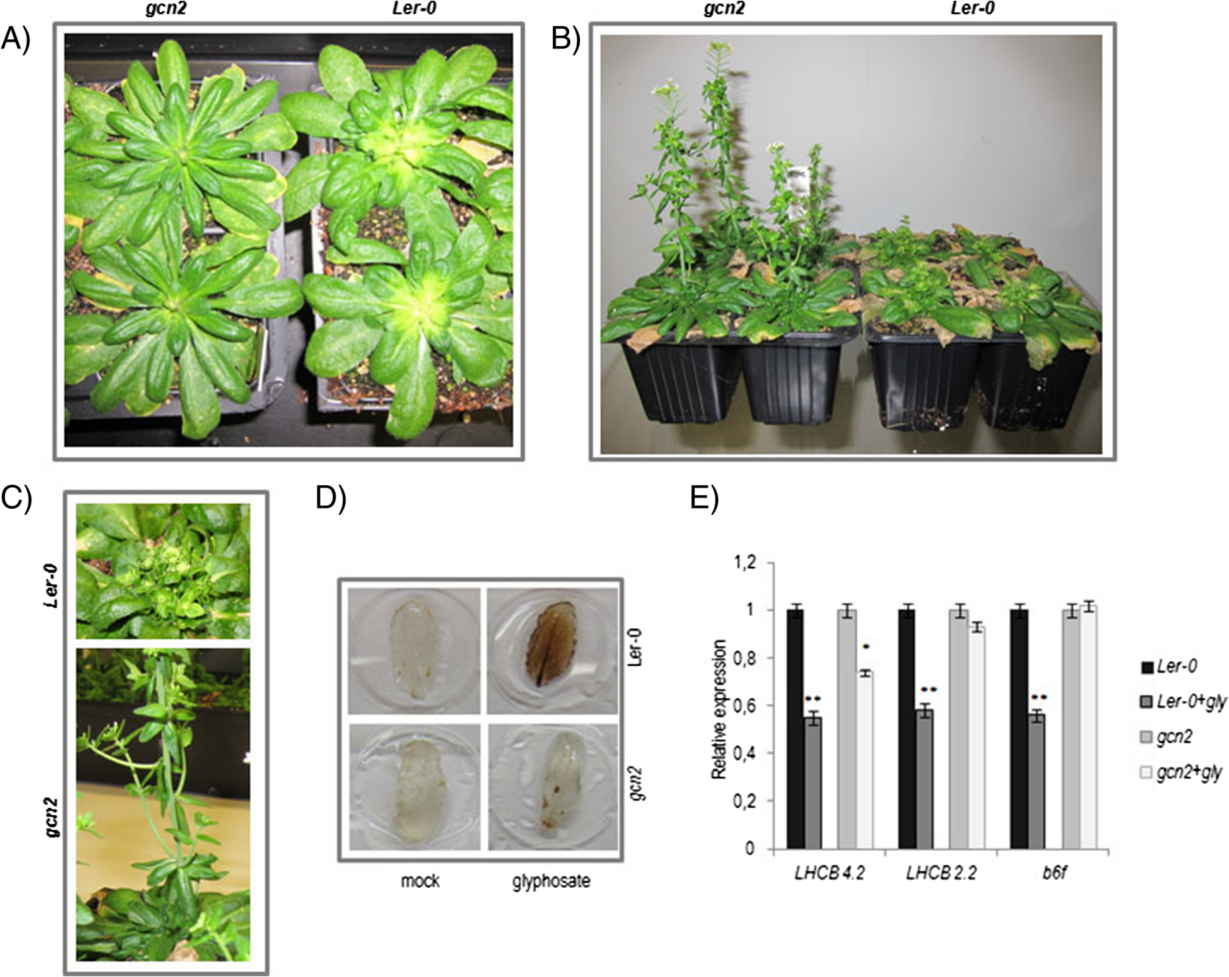
**Glyphosate effects over wild-type and gcn2 four-week old plants.** Pictures show aspect of four-week old glyphosate-treated *Arabidopsis* wild-type (Ler-0) and *gcn2* GT8359 plants, after glyphosate treatment. The experiment was repeated three times using 48 individual plants per genotype in every experiment. Mock-treated plants were looking similar (data not shown). General view of glyphosate-treated plants two-weeks **(A)** and four weeks after treatment **(B)**. **(C)** Close-up of Ler-0 and *gcn2* plants from Figure 1B. **(D)** Apical leaves DAB-staining of mock- and glyphosate-treated plants. Five independent plants were used as biological replicates, and two rosette leaves were sampled from each plant. The experiment was repeated three times. **(E)** Relative transcript levels of *LHCB 4.2* (At3g08940), *LHCB 2.2* (At2g05070), and *B6F* (At5g36120) in wild-type (Ler-0) and *gcn2* plants. Data show mean and standard error of three independent biological replicates. Each replicate contains material from five independent plants (*t*-test, *P < 0.05, **P < 0.01).

Interestingly, more than a thousand genes are differentially expressed in the *gcn2* mutant line after treatment with glyphosate as compared with the wild-type, according to the criteria specified in Methods. Looking at more detail, around 75% of the genes regulated by glyphosate are regulated with the same trend in the *gcn2* plants, but being the fold-change lower in the mutant plants [see Additional files 1 and 3]. That is, a gene induced by glyphosate can still be induced in a *gcn2* mutant, but with a lower fold-change. This effect is more remarkable for those genes with the highest levels of expression. This observation suggests that cellular responses to glyphosate are compromised although not totally abolished in the *gcn2* mutant. The transcriptional repression of the photosynthesis is the biological process most affected by the lack of GCN2, as stated by GO biological processes studies [see Additional file 7 and Table 2]: the expression of genes belonging to photosynthesis processes is significantly higher in *gcn2* plants after glyphosate treatment (1.59×10^−25^ adjusted p-value, Figure 1B), indicating that the repression that takes place in wild-type after glyphosate treatment is also compromised. The same effect is observed for cell division, redox homeostasis and other categories. Equally, among the genes previously shown to be induced by glyphosate that are deregulated in the *gcn2* mutant line are those involved in defense against both biotic and abiotic stimulus. As reported by Zhang et al. [20], our functional categories data show that amino acid biosynthesis is not differentially regulated between wild-type and *gcn2* plants (Additional file 4), although differences in expression were found for specific genes of the pathway (see Additional files 1 and 3).

**Table 2:** **Selected categories enriched in wild-type vs.** ***gcn2*** **Arabidopsis seedlings after glyphosate treatment (for a complete list, see Additional file** **2** **)**

**Selected GO categories enriched in wild-type as compared with gcn2 in glyphosate-induced genes**		**Selected GO categories enriched in** ***gcn2*** **as compared with wild-type in glyphosate –repressed genes**	
**GO biological process**	**Adj. p-value**	**GO biological process**	**Adj. p-value**
Response to wounding	3.45×10^−24^	Photosynthesis	1.59×10^−25^
Response to bacterium	0.0000377	Microtubule-based movement	8.52×10^−12^
Autophagy	0.000157	Chlorophyl biosynthetic process	6.47×10^−8^
Response to salt stress	0.000370	Photosynthesis electron transport	7.53×10^−8^
Response to osmotic stress	0.000402	Cell division	0.0000683
Response to fungus	0.00143	Regulation of cell size	0.001056
Multidrug transport	0.00650	Cell growth	0.0044
Cell death	0.00675	Electron transport chain	0.00716
Response to oxidative stress	0.0184	M phase of cell cycle	0.049

Selected categories enriched in wild-type or *gcn2* plants after an Enrichment Analysis using FatiGo (Medina et al. [51]) on the differentially expresses genes (fold-change > 2 and adjusted p-values < 0.05) in 16-day-old *Arabidopsis* wild-type Landsberg and *gcn2* GT8351 seedlings treated with glyphosate as described in Methods. For a complete list of GO categories (biological process) with an adjusted p-value lower than 0.05 see Additional files 4 and 7.

### *gcn2* plants show less glyphosate-derived effects

The observed dependence on the GCN2 kinase in glyphosate-induced responses was confirmed in adult plants. 200 μM glyphosate was sprayed on four-week-old *Arabidopsis* wild-type and *gcn2* plants and growth over the next three weeks after treatment was followed. Again, *gcn2* plants are phenotypically indistinguishable from wild-type plans at this stage of growth before the treatment (Additional file 5: Figure S2). As observed in Figure 1A, wild-type plants start showing typical phytotoxic glyphosate effects after 12–14 days. Meristems and young tissues became chlorotic and disorganized. In the following weeks, apical dominance was lost and plants presented a characteristic shoot-branching phenotype (Figure 1B and C). Interestingly, these effects were much less severe in *gcn2* plants. Although a subtle sensitivity could be observed the first days after treatment (initial chlorosis at the meristem area, data not shown), we did not observe severe glyphosate effects in *gcn2* plants two-weeks after treatment, when chlorotic tissues were clearly observed in wild-type plants. As shown in Figure 1A, *gcn2* rosettes continued growing during the two weeks after the treatment, although new leaves morphology was more compacted. Later, *gcn2* plants recovered from the stress and were growing again with a less severe branching-phenotype than wild-type plants (Figure 1B-C).

**Figure 2:**
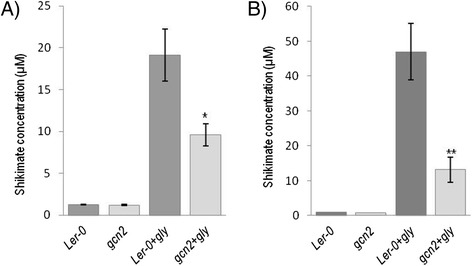
**gcn2 plants accumulate less shikimate than wild-type** ***.*** Quantification of shikimate levels in seedlings **(A)** and adult plants **(B)** 72 h after mock treatment (Ler-0, *gcn2*) or glyphosate treatment (Ler-0 + gly, *gcn2* + gly). Data show mean and standard error of ten independent biological replicates. Asterisks represent significant differences between wild-type and *gcn2* plants (*t*-test, *P < 0.05, **P < 0.01).

To evaluate in more detail the biochemical differences between both genotypes, the effects of glyphosate on various parameters associated with photosynthetic was measured in non-chlorotic healthy leaves two-weeks after treatment, when the first symptoms of the herbicide were already visible in younger tissues. As shown in Table 3 and described previously in other works [30] and references therein, photosynthetic rate, stomatal conductance, transpiration and quantum efficiency of photosystem II were rapidly inhibited in Ler-0 leaves sprayed with glyphosate as compared with mock-treated plants. The increase in substomatal CO_2_ concentration with decreasing stomatal conductance suggests biochemical limitations to photosynthesis. Although no changes in Fv/Fm were observed, the herbicide provoked a significant decrease in chlorophyll content.

**Table 3:** **Effect of glyphosate application on the photosynthetic rate (A**_**N**_ **), stomatal conductance (g**_**s**_ **), substomatal CO**_**2**_ **concentration (C**_**i**_ **), transpiration rate (E), quantum efficiency of photosystem II (PhiPS2), maximum quantum yield efficiency (Fv/Fm) and SPAD index**

**Genotype**		**A**_**N**_ **(μmol m** ^**−2**^ **s** ^**−1**^ **)**	**g**_**s**_ **(mol m** ^**−2**^ **s** ^**−1**^ **)**	**C**_**i**_ **(μmol mol** ^**−1**^ **)**	**E** **(mmol m** ^**−2**^ **s** ^**−1**^ **)**	**PhiPS2**	**Fv/Fm**	**SPAD (a.u.)**
Ler	Control glyphosate	7.3a	0.13a	301b	3.3a	0.106a	0.822	33a
		3.1b	0.05b	328a	2.0b	0.059b	0.826_NS_	27b
*gcn2*	Control glyphosate	7.5	0.10	288a	3.1	0.134	0.834	34b
		7.2_NS_	0.08_NS_	255b	2.6_NS_	0.125_NS_	0.836_NS_	37a

Each value is the mean of eight independent determinations in different plants.

For each genotype, different letters indicate significant differences (*P* < 0.05); NS: not significant.

By contrast, the application of the herbicide has negligible effects on photosynthetic measurements in the *gcn2* mutant plants. No significant differences were observed between mock-treated and glyphosate-treated plants on CO_2_ assimilation, stomatal conductance, quantum efficiency of photosystem II or maximal quantum yield (Table 3). These results further confirm the dependence on GCN2 of the glyphosate-induced repression of photosynthesis.

These results agree with the relative expression of several photosynthetic genes in both genotypes. We analyzed by RT-PCR the expression of the three genes most repressed by glyphosate in the seedlings experiment, namely two light-harvesting complex genes (LHC 2.2 and LHC 4.2) and a gene involved in the assembly of cytochrome b_6_f (At5g36120). These three genes were not repressed in *gcn2* seedlings [see Additional file 3]. As shown in Figure 1E, expression of these genes was repressed in wild-type plants after glyphosate treatment, but the effect was not observed in the *gcn2*-treated plants, confirming the results obtained in seedlings.

DAB staining is used as an efficient method to detect hydroxen peroxide accumulation in plant tissues [31] and H_2_O_2_ increases have been described in plants treated with glyphosate [9]. Young leaves of wild-type plants after glyphosate treatment were stained using DAB. As shown in Figure 1D, a dramatic accumulation of H_2_O_2_ is observed, preceeding the cell death observed in the following days. As expected, *gcn2* plants of the same age did not show H_2_O_2_ accumulation, further confirming the dependence on GCN2 of the oxidative-stress burst observed after glyphosate treatment.

In summary, gene expression data, as well as glyphosate effects such as photosynthesis decay and oxidative stress, suggest that *gcn2* plants are less affected by glyphosate than wild-type plants.

### Shikimic acid accumulation is compromised in *gcn2* plants after glyphosate treatment

Glyphosate inhibition of the enzyme 5-enolpyruvyl-shikimate-3-phosphate synthase leads to reduced feedback inhibition of the pathway, resulting in carbon flow to shikimate-3-phosphate, which is converted into high levels of shikimate [7]. Given that *gcn2* plants were less prone to glyphosate effects, as observed in the experiments described above, we wanted to determine if the target enzyme of glyphosate was inhibited in the same way in both lines. *Arabidopsis* wild-type and *gcn2* seedlings were treated with glyphosate as explained above, and shikimic acid accumulation was measured three days after treatment. The same experiment was performed in four-weeks old plants, in the way explained above and in Methods. As observed in Figure 2, basal levels of shikimic acid are similar in both lines, either in seedlings or in adult plants. However, 3-days after glyphosate application, shikimic acid in wild-type seedlings has increased almost 20 times over the basal levels (46 times in adult plants treatments), whereas *gcn2* only accumulates half the amount of shikimic acid that wild-type does (only 10 times in seedlings, 20 times in adult plants). This trend indicates that the target enzyme of glyphosate is not being inactivated at the same extend in *gcn2* plants, or that the metabolic flux to the pathway has been reduced in *gcn2* plants.

### Glyphosate uptake is not affected in *gcn2* plants

In the plant, EPSPS is encoded in the nucleus and translocated to the chloroplast, where the aromatic amino acids are synthesized [32]. The highest shikimate accumulation occurs in the youngest tissues, suggesting that glyphosate enters the plant and then translocates to the active meristems to reach the target site, where its action takes place preferentially [7]. Several reasons could then account for the less glyphosate action observed in *gcn2* plants. In one hand, glyphosate uptake and/or translocation could be compromised. Less concentration of active glyphosate will be present in the plant chloroplasts, explaining the apparent resistance of *gcn2* plants to the herbicide. On the other hand, detoxification mechanisms could be exacerbated in the mutant, inactivating the herbicide.

The mechanisms of glyphosate uptake into plant cells are not well understood. At least at low concentrations of herbicide (in the micromolar range, as it is used in this study) it seems to involve a phosphate transporter, as glyphosate uptake is inhibited by sodium phosphate and phosphonoformic acid, a competitor inhibitor of phosphate transport in plants [33,34]. In order to ascertain if *gcn2* plants were compromised in phosphate transport, that could confer to these plants an advantage in the presence of the herbicide*,* we performed germination assays of wild-type and *gcn2* seeds in media with phosphate deficiency. No differences were observed between wild-type and *gcn2* seedling when grown in media containing 0-500 μM phosphate (data not shown), indicating that *gcn2* plants are taking up phosphate in a similar way as wild-type does. The same results were observed in root-growth assays (data not shown).

## Discussion

Little is known about the molecular events that contribute to non-target-site based resistance (NTSR), the combination of mechanisms that limit to a non-lethal dose the amount of herbicide reaching the target-site. In this study, we identify GCN2 as a cellular component that fosters the action of glyphosate in the model plant *A. thaliana*. GCN2 is a conserved protein kinase responsible for the phosphorylation of the initiation factor eIF2-α after a number of stress situations. In *Arabidopsis*, herbicide treatments, wounding, cold treatments, UV or purine starvation has been described to activate AtGCN2 [21] the only kinase able to phosphorylate eIF2-α in this species [20]. Phosphorylation of eIF2-α prevents further cycles of protein translation, and it is assumed that this helps the cell to conserve metabolic resources until it has overcome the immediate biological impact of the stress [29]. In this model, the activation of GCN2 should then be beneficial for the plant to cope with the stress. However, we have shown that the presence of GCN2 is somehow facilitating the action of the herbicide, and cellular responses to glyphosate are not triggered or attenuated in a mutant line that is not able to phosphorylate eIF2-α. This is not the first report where the lack of GCN2 is conferring an advantage when a particular stress is applied. In yeast, ScGCN2 acts also as a negative factor, conferring toxic effects on growth under NaCl stress, and being a *gcn2* knock-out strain able to grow normally under 400 mM NaCl [35]. In human tumor cells, HsGcn2 was shown to have an unexpected proapoptotic effect under glucose deficiency stress, and *gcn2* knock-out cells are able to survive more than wild-type under these stress conditions [36]. As such, these stresses and the canonical amino acid starvation may utilize distinct pathways that converge on eIF2α phosphorylation with opposing biological outcomes [36]. It will be interesting to know whether a *gcn2* background confers resistance to other abiotic stresses in *Arabidopsis*, and to investigate if this resistance is converging in a single factor for all stresses or in different factors for every one of them.

Known responses to glyphosate include the rapid repression of photosynthesis. Inhibition of CO_2_ assimilation and depletion of intermediates of the carbon reduction cycle had been documented years ago [37] and these effects had been attributed to an upregulated flux into the shikimate pathway due to depletion downstream of EPSPs. Recent transcriptomic assays revealed that this repression could be also genetically regulated. In a comparative study of *Festuca* species, Cebeci and Budak reported that, 5 days after treatment with glyphosate, a marked repression of photosynthetic genes, including chlorophyll biosynthesis, photosystems and Calvin cycle enzymes was occurring [14]. Consistent results were obtained in proteomic assays performed in rice [11]. We have also observed repression in gene expression in adult plants 15 days after treatment, consistent with a clear inhibition of photosynthetic rate. The dramatic repression of photosynthetic genes only 6 h after treatment to *Arabidopsis* seedlings, when no visible symptoms of leaf chlorosis were observed yet (Table 1), suggests that besides a likely photosynthetic decrease due to metabolic toxicity, an early genetically programmed inhibition of photosynthesis is also taking place. Consistent with our data, repression in some photosynthetic genes was observed in soybean sensitive plants only 4 h after treatment with glyphosate [15]. This decay of photosynthesis after glyphosate treatment is not observed in *gcn2* plants, nor is the oxidative stress that characterizes the herbicide effect (Table 2). Moreover, enzymes known to be involved in the detoxification of herbicides such as ABC transporters, glutathione-transferases or glycosyltransferases [38,39] are dramatically activated after glyphosate treatment in wild-type plants, but unaltered or weakly activated in *gcn2* plants [see Additional file 1]. Finally, shikimate accumulation in *gcn2* plants compared to wild-type clearly demonstrates that the lack of GCN2 becomes an advantage when plants are treated with this herbicide.

At present, these data do not allow to discern at what point the herbicide action benefits from the presence of GCN2 in the cell. Glyphosate is said to starve cells of aromatic amino acids, due to inhibition of the shikimate pathway and the production of chorismate, the precursor of phenylalanine, tryptophan and threonine. However, total amino acid pools after glyphosate treatment rather increase than decrease [40,41] after the first days after treatment, as a consequence of the induction of some proteolytic activities [10] or are not much different in glyphosate resistant soybean as compared to sensitive cultivars [29]. The known mechanism of activation of GCN2 through uncharged t-RNAs [18] also works in *Arabidopsis* [42] and although not described in detail for glyphosate, treatment with chlorsulfuron, that blocks valine, leucine and isoleucine biosynthesis [20] yield a peak of eIF2-α phosphorylation 6 h after treatment but return to basal levels after 24 h. Starvation of amino acids and prolonged protein translational arrest via GCN2 are then unlikely to be the major cause of the slow effects of glyphosate treatment. The involvement of GCN2 in the glyphosate mode of action should then fall on the first hours after the treatment, this conditioning the final effect in the plant. How could GCN2 foster the action of the herbicide? As stated above, the early activation of the kinase in wild-type plants after glyphosate application, likely due to the initial decay in aromatic amino acids, do not avoid the expression of cellular factors involved in detoxification of xenobiotics. In some species, vacuolar sequestration is contributing to the resistance mechanism in resistant variants [43]. If the same mechanism is working in *Arabidopsis,* activation of GCN2 could impair vacuolar membrane trafficking through inhibition of some important protein. Alternatively, the selective translation of certain mRNAs with uORFs in the leader sequence, in the same way of *ScGCN4*, in yeast, and *HsATF4*, in humans [18], could facilitate translocation of glyphosate to the young tissues, where the target enzyme is mainly expressed and the action of glyphosate is more dramatic [7]. Reduced translocation to meristematic sinks is a major mechanism of resistance in horseweed [44], Italian ryegrass [45] or hairy fleabane [46]. However, no homologous sequence of *ScGCN4* has been found in Arabidopsis so far, and a GCN2-dependent selective translation of mRNAs is unknown. Finally, the absence of GCN2 activity in the mutant *gcn2* line could provide a constitutive advantage in the mutant background that diminished herbicide effects, independently of the post-treatment GCN2 activation. One possibility was a higher uptake of glyphosate in wild-type plants. Although the mechanism of glyphosate uptake into plant cells is not well understood, the involvement of a phosphate transporter has been proposed [32,47]. *gcn2* plants are not more sensitive than wild-type to growth in phosphate deficiency media, and microarray experiments over non-treated seedlings [see Additional file 1] did not reveal differences in gene expression that make suspect of phosphate transport missregulation, indicating that phosphate transport is not compromised in *gcn2* plants. If phosphate transporters are involved in glyphosate uptake in *Arabidopsis*, then *gcn2* plants should be taking up the herbicide at the same rate as wild-type plants.

Several attempts have been made to find genes involved in glyphosate resistance using mutant collections of *A. thaliana* [48,49]. In Brotherton et al., the same concentration of glyphosate used in this study was used in a germination assay to find EMS-mutagenized mutant lines of *Arabidopsis* resistant to the herbicide, but no resistant mutant was recovered [48]. If glyphosate resistance single mutations were common, they should have been found in these saturation mutagenesis studies [47]. The lack of *GCN2* does not confer resistance to glyphosate in germination assays (data not shown). In a seedling assay performed by Zhang et al., the *gcn2* mutant line showed sensitivity to glyphosate treatment [20]. However, we were not able to find this sensitivity in a similar experiment using plants in the same developmental stage (data not shown). May be subtle differences in the experimental conditions are the cause of this apparent discrepancy. A *gcn2* knock-out mutant conferring resistance or sensitivity to the same stress has been already reported in animal cell cultures, GCN2 acting as a molecular switch that shifts cells from a proapoptotic to a cytoprotective state in response to glucose deficiency [35], depending on the duration of the stress. In our experimental setup, we have shown that shikimate is not accumulating and gene activation is not being triggered at the same rate than in wild-type plants, indicating that this protein kinase could be an important clue to discover components involved in the resistance to this herbicide. “Glyphosate is as important to world agriculture as penicillin is to human health” stated Stephen Powles, director of the Australian Herbicide Resistance Initiative (http://www.ahri.uwa.edu.au). Given the spread of glyphosate resistance weeds around the planet and the economic importance for agriculture, understanding the mechanisms of such resistance could help to design new biotechnological approaches for a more efficient use of this important herbicide.

## Conclusions

Several mechanisms have been proposed for non-target based resistance to glyphosate, but any study has so far identified any gene that could be directly involved or influencing the final effect of this herbicide in the plant. We have demonstrated that the translational regulator GCN2 is fostering the action of the herbicide by an unknown mechanism. The loss-of-function *gcn2* mutant in the model plant *A. thaliana* emerge as an important tool to decipher the way glyphosate enters the plant and reach its target site. This information will help to design new strategies to preserve the use of glyphosate in the emerging glyphosate-resistance-weeds era.

## Methods

### Plant growth and treatments

*A. thaliana* accession Landsberg erecta (Ler-0) was used in this study. Genetrap GT8359, containing a Ds transposable element interrupting the *GCN2* gene [20] was obtained from Cold Spring Harbor Laboratory, New York (http://genetrap.cshl.edu/).

Seeds were pretreated in 70% ethanol for 20 min, surface-sterilized in 2.5% bleach for 10 min, and washed with distillated water at least five times. After stratification at 4°C in the dark for 5 days, seeds were sown on 1% agar-containing MS Salts, 1% sucrose, pH 5.5, and grown at 23°C with a 16-h-light/8-h-dark cycle.

For experiments at seedling stage, 16-day-old plantlets (see Additional file 5: Figure S2 for pictures) were submerged in 200 μM glyphosate (SIGMA) or distilled water (mock) for 1 minute, and incubated for further growth on liquid MS medium, 1% sucrose, pH 5.5, under the same conditions. Samples for microarray experiments were collected 6 h after treatment. Samples for shikimate assays were collected 72 h after treatment. Visual inspection was observed during the next two weeks after treatment.

For experiments in adult plants, 10 to 15-day-old plantlets were transferred to soil and grown at 23°C under short-day conditions (8-h-light/16-h-dark). Glyphosate treatments were done three-weeks later (see Additional file 5: Figure S2 for pictures). Plants were sprayed with 200 μM glyphosate once (or distilled water for mock treatments), using a standard sprayer by applying three pulses to every plant at a distance of 10–15 cm from the rosette (aprox 250 μL per plant), and incubated for further growth under the same conditions. Expression analysis by RT-PCR, DAB staining and photosynthetic measurements were done two-weeks after treatment. Visual inspection was followed during the next four weeks after treatment.

### Microarray experiments

Total RNA of glyphosate- and mock-treated 16-day-old seedlings was extracted using RNeasy kit (Qiagen). 1.5 μg of total RNA was labeled using MessageAmp II amplification kit from Life Technologies, following manufacturer instructions. Labeling was done using Cy3 and Cy5 dyes from GE (RPN5661). Before hybridization, slides were pre-hybridized at 42°C for 45 m in 5xSSC, 0.1%SDS and 0.1 mg/mL BSA. Microarray hybridizations and washings were done in manual chambers at 42°C, according to Forment et al. [50]. Scanning and Image Analysis was performed using GenePix Pro 6.0 software (Molecular Devices).

For wild type vs. wild type + Glyphosate, two biological replicates were done. Expression ratios in both microarrays were averaged and considered for further analysis if equal trend (induction or repression) was observed in both replicates. Gene set Enrichment Analysis was done using Fatiscan [51] taking as significant those categories with and adjusted p-value lower than 0.05.

For *gcn2* mutant + Glyphosate vs. wild type + Glyphosate, three biological replicated were done swapping the dyes in one of them. A gene was considered differentially expressed if average fold-change was higher than 2 and had a FDR < 5% after a SAM test [52]. Functional analysis was done using FatiGO [51] taking as significant those categories with and adjusted p-value lower than 0.05. The same analysis was performed for *gcn2* mutant vs. wild type without glyphosate.

These microarrays data have been included in the GEO Omnibus database with the reference numbers GSE 56146 and GSE 56147.

### Shikimate assay

Shikimate determination was done 3 days after treatment in seedlings and 7 days after treatment in adult plants. For the seedling assay, fresh weight was annotated before freezing. From each adult leaf, three discs (4 mm diameter using a micropunch) were placed in a 2-ml tube. Seedlings and discs were frozen with liquid nitrogen and kept at −80 until use. Extraction of shikimate was performed as described in Koger et al. [53]. Vials were removed from the freezer and 0.25 M HCl was added to each vial (1 ml per 100 mg FW or 100 μl per leaf disc). Vials were mixed by vortexing and incubated at room temperature for 1.5 h. Afterwards the solution was frozen and kept at −20°C until analysis. Shikimate was analyzed by HPLC as described before [40].

### Photosynthetic measurements

Gas exchange and chlorophyll fluorescence measurements were performed as described by Flexas et al. [54]. Instantaneous determinations of net CO_2_ assimilation rate (A_N_), stomatal conductance (g_s_), transpiration rate (E) and substomatal CO_2_ concentration (C_i_) were carried out at steady-state conditions under saturating light (1000 μmol m^−2^ s^−1^), a vapour pressure difference (vpd) between 1 and 2 kPa and 400 ppm CO_2_ with a LI-6400 (LICOR, Nebraska, USA). The actual photochemical efficiency of photosystem II (PhiPS2) was determined by measuring steady-state fluorescence (F_s_) and maximum fluorescence (F_m_’) during a light-saturating pulse (8000 μmol m^−2^ s^−1^) [55]. Maximal photochemical efficiency (F_v_/F_m_) on dark adapted leaves was measured with a MINI PAM fluorometer (Walz, Effeltrich, Germany). SPAD values were measured with a chlorophyll meter SPAD-502 (Konica Minolta, Osaka, Japan). One measurement per plant was taken on the 8^th^ to 10^th^ leaf from the apex, and for each genotype and treatment, 8 different plants were measured.

### DAB staining

In situ detection of hydrogen peroxide was performed by staining with diaminobenzidine (DAB) staining, according to Daudi et al. [31] with modifications. Briefly, rosette leaves were incubated in staining buffer (1 mg/mL DAB containing Tween 20 (0.05% v/v) and 50 mM sodium phosphate buffer (pH 3.8) and vacuum infiltrated applying 3 pulses of 1.5 m, and stained for 24 h at room temperature. Leaves were fixed in ethanol:glycerol:acetic acid 3:1:1 (bleaching solution) placed in a water bath at 95°C for 15 m. Bleaching solution was replaced and plants were visualized under white light and photographed.

### Real-time PCR

For RT-PCR experiments, total RNA was extracted using RNeasy kit (Qiagen) and treated with DNase I to remove genomic DNA. cDNA was obtained using the Maxima First Strand cDNA Synthesis Kit (Fermentas). Quantitative real-time PCR was performed in a 7500 Fast Real-Time PCR System, from Applied Biosystems, using EvaGreen as a fluorescent reporter and Taq polymerase (Biotools). Primers were designed using PRIMER3 software. Actin 8 (At1g49240) was used as an internal control (Fw 5′-AGTGGTCGTACAACCGGTATTGT; Rv 5′- GAGGATAGCATGTGGAAGTGAGAA). Primers for LHCB 4.2 (Fw 5′- CCACTCTTGGCGCTATCAC; Rv 5′- GCCGATCACTAACACTTCGAT). Primers for LHCB 2.4 (Fw 5′- AGCGACCTCATCCAAAAGG; Rv 5′- TCCGAGAATGGTCCCAAGTA). Primers for B6F (Fw 5′- AGTGACCACCAGCTTCGTCT; Rv 5′- AAGAGACGTGGATCGATTGC). The reaction commenced at 95°C for 5 m, followed by 40 cycles of 15 s at 95°C, 30 s at 55°C, and 30 s at 72°C. Data were analyzed using 7500 Applied Biosystem proprietary software v.2.0.4.

### Availability of supporting data

The data sets supporting the results of this article are available in the GEO repository (GSE56146 and GSE56147).

## Additional files

Additional file 1:
**File showing genes induced by glyphosate in 16-day-old Arabidopsis wild-type Landsberg and in** ***gcn2*** **mutant.**
Additional file 2:
**GO categories (biological process) enriched in 16-day-old Arabidopsis wild-type Landsberg seedlings treated with glyphosate compared with mock-treated plants.**
Additional file 3:
**File showing genes repressed by glyphosate in 16-day-old Arabidopsis wild-type Landsberg seedlings and in** ***gcn2*** **mutant.**
Additional file 4:
**GO categories (biological process) enriched in 16-day-old Arabidopsis wild-type Landsberg seedlings treated with glyphosate compared with treated** ***gcn2*** **plants.**
Additional file 5: Figure S2. Pictures showing a comparison of wild-type (Ler) and *gcn2* plants at the seedling stage (16-day-old) grown in MS plates (A), at three-weeks old plants (B) and four-weeks old plants (C). White bar indicates 1 cm. Additional file 6: Figure S1. Western blot showing inmunodetection of phosphorylated eIF2α in protein extracts of Arabidopsis seedlings used for microarray experiments (upper panel). Coomassie staining of a band of 45 kDa (lower panel) was used as a loading control. Additional file 7:
**GO categories (biological process) enriched in 16-day-old Arabidopsis** ***gcn2*** **seedlings treated with glyphosate compared with wild-type-treated plants.**
