# Supplementary figures and images for: Protein kinase GCN2 mediates responses to glyphosate in Arabidopsis
Source: BMC Plant Biol. 2015 Jan 21;15:14. doi: 10.1186/s12870-014-0378-0 (PMC4312595; doi:10.1186/s12870-014-0378-0)

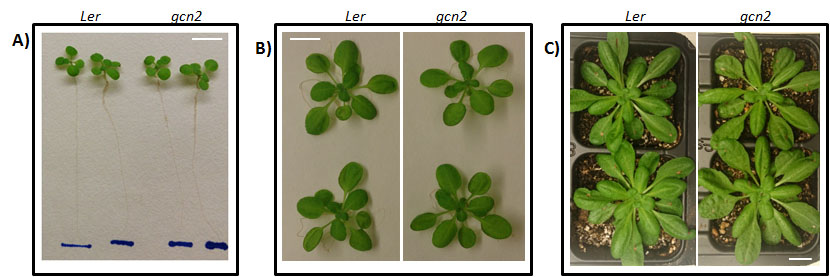

Supplement: Additional file 5: Figure S2. — Pictures showing a comparison of wild-type (Ler) and gcn2 plants at the seedling stage (16-day-old) grown in MS plates (A), at three-weeks old plants (B) and four-weeks old plants (C). White bar indicates 1 cm. [file 12870_2014_378_MOESM5_ESM.jpeg]

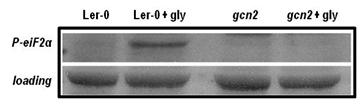

Supplement: Additional file 6: Figure S1. — Western blot showing inmunodetection of phosphorylated eIF2α in protein extracts of Arabidopsis seedlings used for microarray experiments (upper panel). Coomassie staining of a band of 45 kDa (lower panel) was used as a loading control. [file 12870_2014_378_MOESM6_ESM.jpeg]
